# A secure heterogeneous mobile authentication and key agreement scheme for e-healthcare cloud systems

**DOI:** 10.1371/journal.pone.0208397

**Published:** 2018-12-12

**Authors:** Han-Yu Lin

**Affiliations:** Department of Computer Science and Engineering, National Taiwan Ocean University, Keelung, Taiwan; King Saud University, SAUDI ARABIA

## Abstract

Heterogeneous mobile authentication is a crucial technique to securely retrieve the resource of e-healthcare cloud servers which are commonly implemented in a public key Infrastructure (PKI). Conventionally, a mobile data user can utilize a self-chosen password along with a portable device to request the access privilege of clouds. However, to validate the membership of users, a cloud server usually has to make use of a password table, which not only increases the burden of management, but also raises the possibility of information leakage. In this paper, we propose a secure heterogeneous mobile authentication and key agreement scheme for e-healthcare cloud systems. In our system structure, an e-healthcare cloud server of traditional PKIs does not have to store a password table. A legitimate data user only possesses a security token hardware and keeps an offline updatable password without using any private key. Our scheme is classified into the category of dynamic ID authentication techniques, since a data user is able to preserve his/her anonymity during authentication processes. We formally prove that the proposed mechanism fulfills the essential authenticated key exchange (AKE) security and owns lower computational costs. To further ensure the practical application security, an automatic security validation tool called AVISPA is also adopted to analyze possible attacks and pitfalls of our designed protocol.

## Introduction

In an open environment such as the Internet, the data communication security is considered as an important issue and has to be ensured to prevent exposure of confidential messages. Whenever a patient tries to request the service of a remote e-healthcare cloud server, the latter must confirm his/her identity before approving the request. We thus call such a protocol the user authentication scheme. Generally speaking, according to the used evidence, we can classify user authentication schemes into the following three techniques:

1. Something you know: It is a kind of intangible knowledge. That is, a user can be authenticated if he proves that he learns something, e.g., password or PIN.
2. Something you have: This approach depends on some tangible objects. A requested user has to reveal some physical objects, (e.g., key, security token or smart card) satisfying the authentication criteria.
3. Something you are: A potential user can only be authenticated if he demonstrates that he owns certain valid biometrical property such as fingerprint, iris pattern and hand geometry.

When a user authentication scheme is combined with two of the above techniques, we call such a protocol two-factor authentication. To protect subsequent data transmission, a shared session key between two parties is often generated after the authentication is achieved. In 1976, Diffie and Hellman [1] proposed the first public key exchange protocol using the hardness of Discrete Logarithm Problem (DLP). Yet, their scheme is easily subject to the man-in-the-middle attack and lacks of user anonymity. Lamport [2] further introduced a password-based user authentication scheme suitable for insecure communication in 1981. In his scheme, a remote server keeps a password table storing hashed passwords rather than plaintexts. However, several later literatures [3–6] still exhibit that his scheme has several security flaws.

To guarantee the characteristic of user anonymity during interactions, in 2004, Das *et al*. [7] addressed the notion of dynamic ID authentication schemes. In such a scheme, a pseudo identity (also known as dynamic ID) of the user is used for interactive authentication processes. It is feasible for a remote server to derive the real identity from a pseudo one, but is computationally infeasible for any adversary to do it. However, their scheme failed to withstand several active attacks pointed by [8–10].

By extending Wang *et al*.’s protocol [9], in 2011, Khan *et al*. [11] proposed an efficient variant which removes the necessity of maintaining a password table. Considering the authentication technique of something you have, Tsai *et al*. [12] incorporated smart cards into his designed protocol. A smart card is usually equipped with lightweight computing capability and limited storage space. Nevertheless, the information stored in the smart card must be carefully selected, or else a malicious adversary can easily obtain the confidential data from a stolen or lost smart card.

In 2011, Wen and Li [13] presented an improved dynamic ID-based remote user authentication with key agreement scheme fulfilling the requirement of user anonymity and supporting the feature of key-update. Unfortunately, in 2012, Tang and Liu [14] found out that their scheme are still vulnerable to known existential attacks. Utilizing the RSA cryptosystem, in 2013, Lin [15] proposed a dynamic ID-based authentication scheme designed for telecare medical information system. He also proved that a previous related work [16] cannot achieve the security requirement of user anonymity and is subject to both dictionary and smart card loss attacks.

In 2014, Chen *et al*. [17] separately demonstrated security flaws of Song’s [18], Sood *et al*.’s [19] and Xu *et al*.’s [20] protocols and gave an enhanced one. Based on elliptic curve cryptosystems, in 2015, Yang *et al*. [21] addressed a two-party authentication key exchange protocol for mobile environments. Using biometric properties, in 2017, Kumari *et al*. [22] came up with a biometrics-based authenticated key agreement scheme for multi-servers. So far, there have been several related variants [4, 23–36] introduced.

Nevertheless, existing schemes are either vulnerable to known attacks, or unsuitable for heterogeneous application environments. This motivates us to design a theoretically and experimentally secure heterogeneous mobile authentication and key agreement protocol in this paper. Particularly, we consider commonly deployed e-healthcare cloud services where a central cloud server of public key infrastructures (PKIs) is responsible for handling requests from various data users of no pre-distributed keys. In our system architecture, we focus on the private cloud environment [37], as many existing hospitals already have their own data centers and essential firewall infrastructures. In this circumstance, hospitals usually bear the most responsibility of managing and securing patients’ medical data. Since the user privacy is another critical point in private clouds, we must be aware of any improper administration that could possibly result in privilege creep [38]. In addition, some general security principles and practices are also helpful for preserving user privacy, including separation of privilege principle, least privilege principle and defense in depth principle. In our protocol design, we adopt dynamic ID authentication techniques to ensure user privacy as well as anonymity. In case that a user’s portable security token device is lost or tampered, it would cause no harm to the user’s privacy, as no confidential data are stored in the form of plaintext.

## Proposed scheme

We demonstrate the proposed heterogeneous mobile authentication and key agreement scheme for e-healthcare cloud systems in this section. Table 1 first defines some utilized symbols for roles, functions, numbers and operations. Without loss of generality, our scheme can be divided into three phases including User Registration, Authentication, and Password-Update. Let *p* and *q* be two large primes satisfying *q* | (*p*– 1) and *g* a generator of order *q*. There are two collision-resistant one-way hash functions, *H*_1_ and *H*_2_, which can accept a variable length input and return an output of fixed length. The notations *ID*_*i*_ and *ID*_*Sj*_ separately represent the identity of a patient *U*_*i*_ and a remote e-healthcare cloud server *S*_*j*_. Detailed steps of each phase are described as follows:

**Table 1:** Symbol notations.

Notation	Description
*U*_*i*_	a patient
*S*_*j*_	an e-healthcare server
*p*, *q*	large primes
*g*	a generator of order *q*
*ID*_*i*_	the identity of *U*_*i*_ and |*ID*_*i*_| = *n*
$ID_{S_{j}}$	the identity of *S*_*j*_ and $\left|ID_{S_{j}}\right|=n$
(*a*)_*n*_	the first *n* bits of the value *a*
*H*_1_(•)	a secure one-way hash function and | *H*_1_(•) | = *n*
*H*_2_(•)	a secure one-way hash function and | *H*_2_(•) | = 2*n*
*PW*_*i*_	*U*_*i*_’s password
*SC*_*i*_	a security token hardware of *U*_*i*_
*x*_*j*_	*S*_*j*_’s private key
*Y*_*j*_	*S*_*j*_’s public key such that $Y_{j}=g^{x_{j}}\mathrm{mod}p$
*k*_*i*_, *a*	random integers
*t*_1_	a timestamp
*SK*	a session key
⊕	the exclusive-OR operation
||	the concatenation operation

### User registration phase

**Fig 1:**
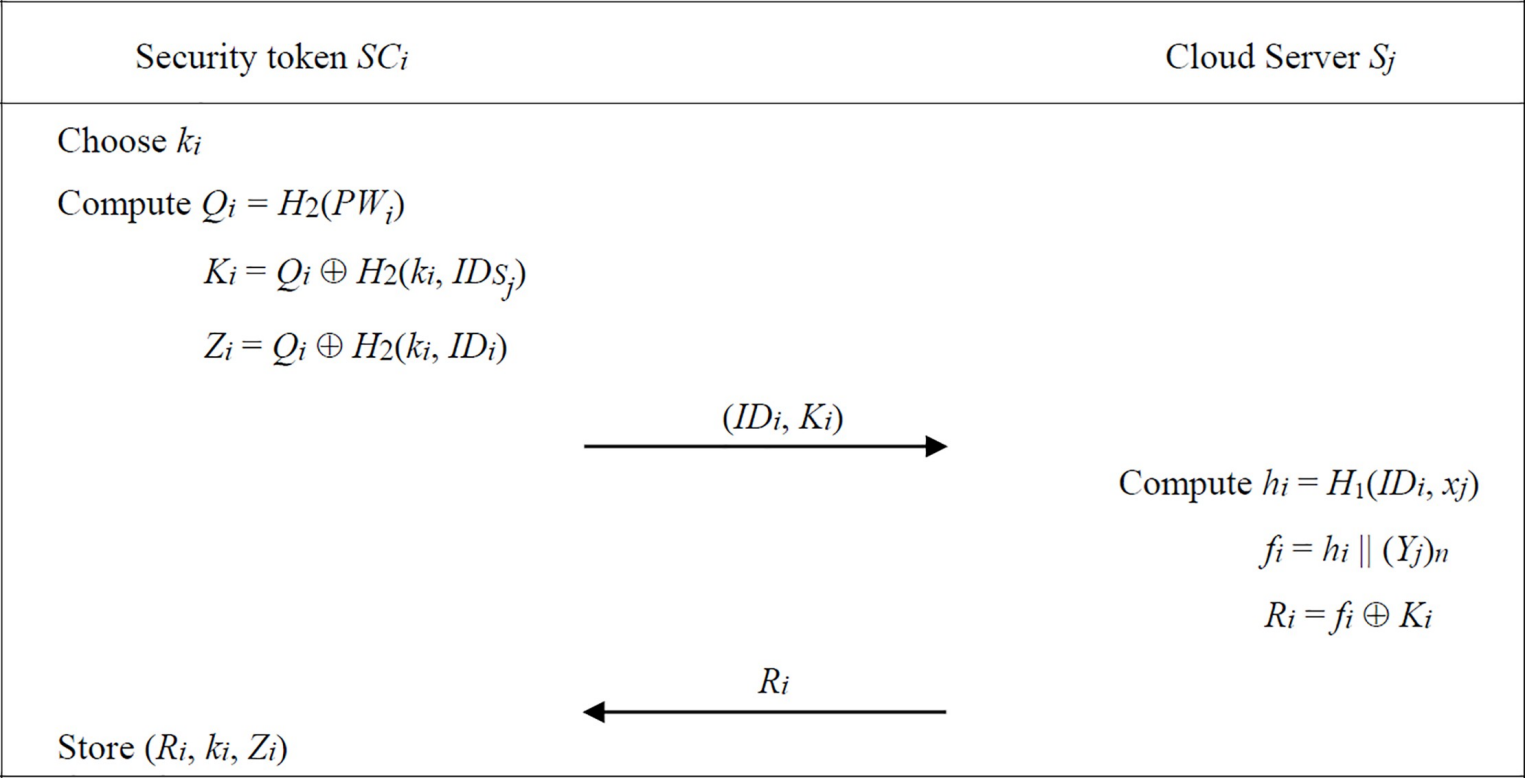
User registration phase of proposed scheme.

Fig 1 illustrates the user registration phase of proposed scheme. Assume that each patient *U*_*i*_ owns a self-chosen password *PW*_*i*_ and a security token device *SC*_*i*_. Before requesting the cloud services from the server *S*_*j*_, *U*_*i*_ has to perform the user registration process for becoming a legitimate user. Initially, *U*_*i*_ will enter his (*ID*_*i*_, *PW*_*i*_) and the *SC*_*i*_ performs the following steps with *S*_*j*_:

**Step 1** *SC*_*i*_ first chooses a random integer *k*_*i*_ to compute *Q*_*i*_ = *H*_2_(*PW*_*i*_) and
  $$K_{i}=Q_{i}⊕H_{2}(k_{i},ID_{S_{j}}),$$ (1)
  $$Z_{i}=Q_{i}⊕H_{2}(k_{i},ID_{i}).$$ (2)
  Then *SC*_*i*_ delivers (*ID*_*i*_, *K*_*i*_) to *S*_*j*_ via a secure channel. Since a random integer *k*_*i*_ is used in computing *K*_*i*_ and *Z*_*i*_, it would be difficult for any malicious adversary to correlate one requested user with another.

**Step 2** After receiving the registration request, *S*_*j*_ computes

$$h_{i}=H_{1}(ID_{i},x_{j}),$$ (3)

$$f_{i}=h_{i}\|{\left(Y_{j}\right)}_{n},$$ (4)

$$R_{i}=f_{i}⊕K_{i},$$ (5)

and returns *R*_*i*_ to *SC*_*i*_ via the same secure channel. *SC*_*i*_ will complete the registration process by storing (*R*_*i*_, *k*_*i*_, *Z*_*i*_). Note that there is no identity related information kept in the *SC*_*i*_. Thus, any attacker cannot have the knowledge of the owner of a lost security token device.

### Authentication phase

**Fig 2:**
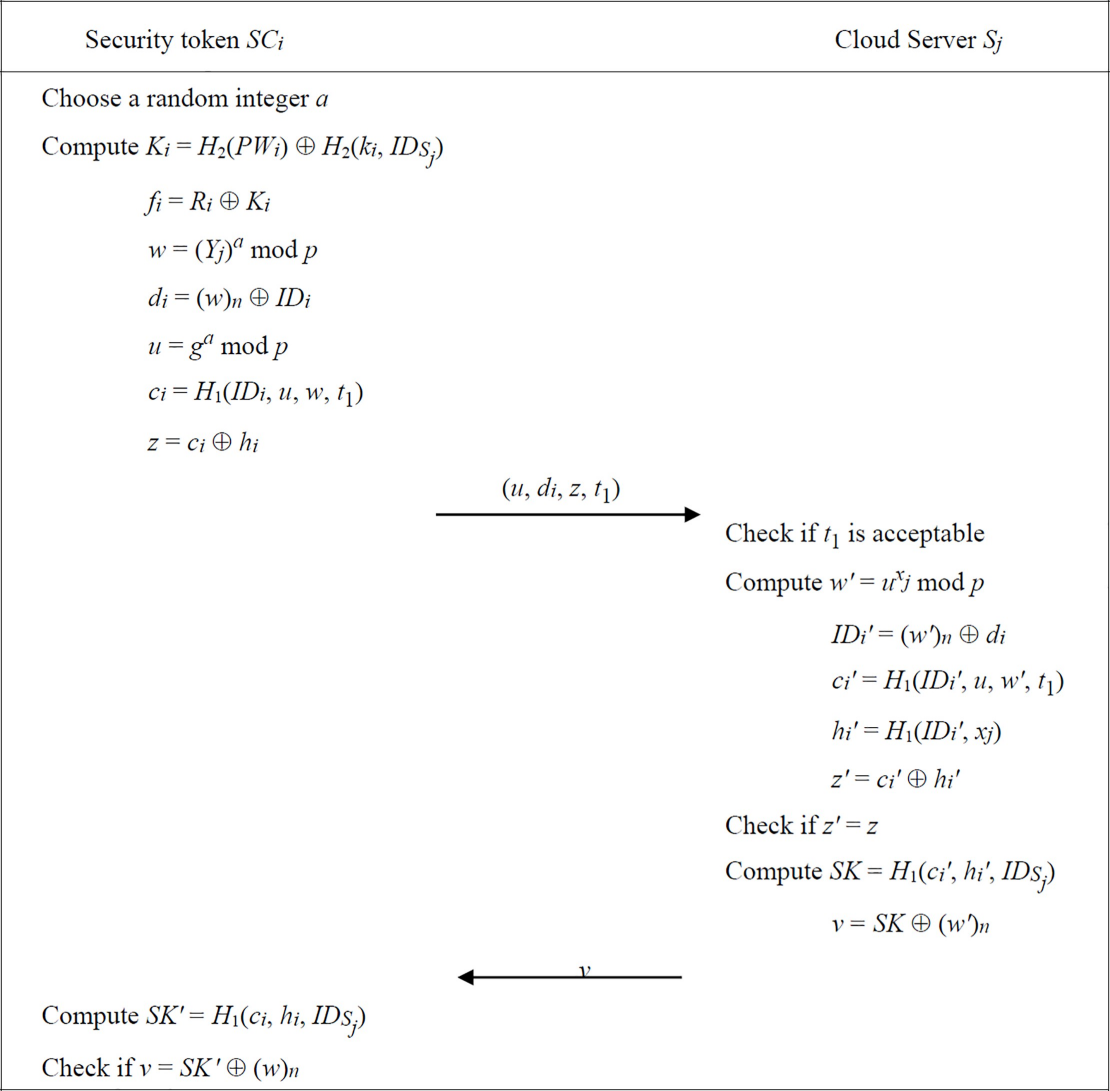
Authentication phase of proposed scheme.

We demonstrate the interactive authentication processes of our scheme in Fig 2. A registered patient *U*_*i*_ can login to remote cloud servers with the assistance of his password and a security token hardware. First, *U*_*i*_ enters his password *PW*_*i*_ and the identity *ID*_*Sj*_ of remote server, and then the security token *SC*_*i*_ will select a random integer *a* and compute necessary parameters as follows:

$$K_{i}=H_{2}(PW_{i})⊕H_{2}(k_{i},ID_{S_{j}}),$$ (6)

$$f_{i}=R_{i}⊕K_{i},$$ (7)

$$w={\left(Y_{j}\right)}^{a}\;\mathrm{mod}\;p,$$ (8)

$$d_{i}={\left(w\right)}_{n}⊕ID_{i},$$ (9)

$$u=g^{a}\;\mathrm{mod}\;p,$$ (10)

$$c_{i}=H_{1}(ID_{i},u,w,t_{1})$$ (11)

$$where\;t_{1}\;is\;a\;timestamp,$$ (12)

$$z=c_{i}⊕h_{i}.$$ (13)

A valid login request is formed by (*u*, *d*_*i*_, *z*, *t*_1_) which is then sent to *S*_*j*_. Upon receiving it, *S*_*j*_ first verifies the freshness of timestamp *t*_1_. If it is not fresh, *S*_*j*_ will deny the service request; else, *S*_*j*_ further computes

$$w^{\prime }=u^{x_{j}}\;\mathrm{mod}p,$$ (14)

$$ID_{i}^{\prime }={\left(w^{\prime }\right)}_{n}⊕d_{i},$$ (15)

$$c_{i}^{\prime }=H_{1}(ID_{i}^{\prime },u,w^{\prime },t_{1}),$$ (16)

$$h_{i}^{\prime }=H_{1}(ID_{i}^{\prime },x_{j}),$$ (17)

$$z^{\prime }=c_{i}^{\prime }⊕h_{i}^{\prime },$$ (18)

and then checks whether *z*′ = *z*. If it holds, *S*_*j*_ derives

$$SK=H_{1}(c_{i}^{\prime },h_{i}^{\prime },ID_{S_{j}}),$$ (19)

$$v=SK⊕{\left(w^{\prime }\right)}_{n},$$ (20)

and sends the confirmation value *v* back to *SC*_*i*_.

Upon receiving it, *SC*_*i*_ also computes

$$SK^{\prime }=H_{1}(c_{i},h_{i},ID_{S_{j}}),$$ (21)

and confirms whether *v* = *SK*′⊕(*w*)_*n*_. If it holds, the user authentication process is successful and the session key *SK* is used for ensuring the confidentiality of this connection.

### Password-update phase

**Fig 3:**
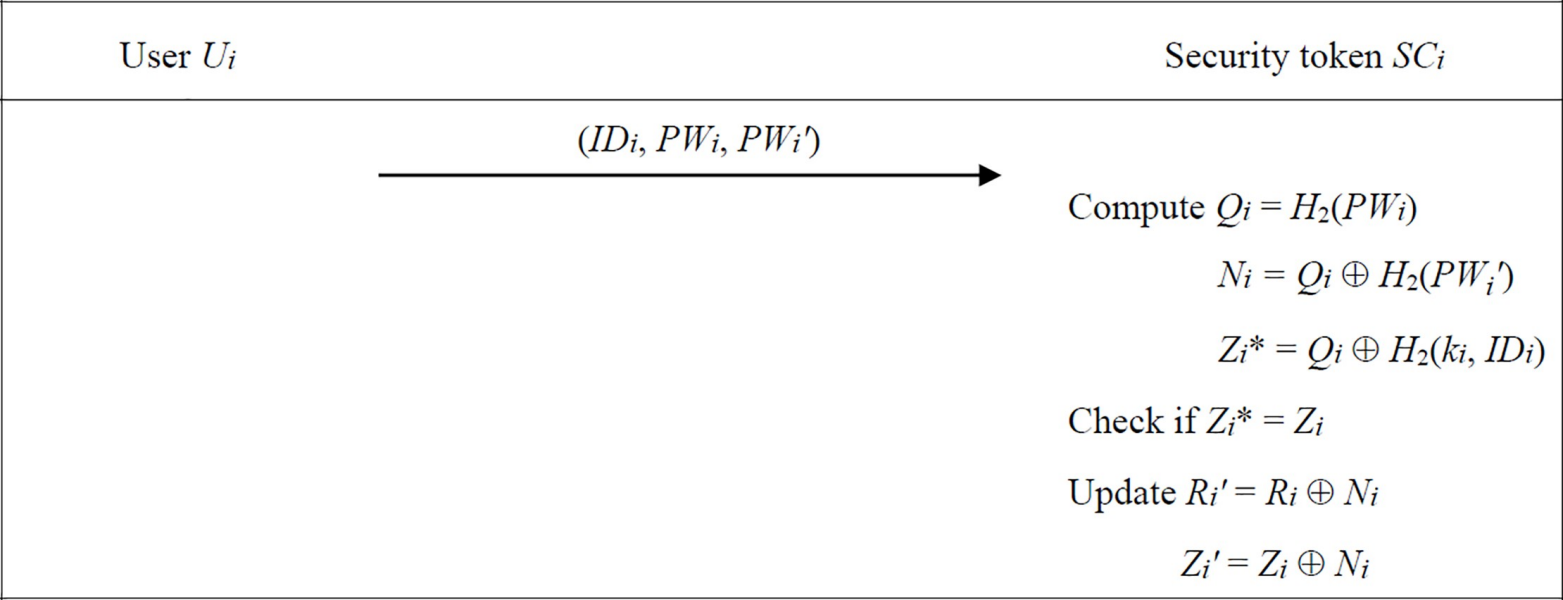
Password-update phase of proposed scheme.

We illustrate the password-update phase of proposed scheme in Fig 3. Each *U*_*i*_ can periodically update his password with his security token device. Note that we do not discuss the issue of password reset or recovery here. Specifically, *U*_*i*_ first enters *ID*_*i*_ along with his old and new passwords (*PW*_*i*_, *PW*_*i*_*'*). Then the security token *SC*_*i*_ computes *Q*_*i*_ = *H*_2_(*PW*_*i*_),

$$N_{i}=Q_{i}⊕H_{2}(PW_{i}^{\prime }),$$ (22)

$$Z_{i}*=Q_{i}⊕H_{2}(k_{i},ID_{i}),$$ (23)

and checks whether *Z*_i_* = *Z*_*i*_. If it holds, *SC*_*i*_ completes the password-update process by modifying the pre-stored *R*_*i*_ and *Z*_*i*_ as

$$R_{i}^{\prime }=R_{i}⊕N_{i},$$ (24)

$$Z_{i}^{\prime }=Z_{i}⊕N_{i},$$ (25)

## Security proof

For facilitating the security proofs of our proposed heterogeneous mobile authentication and key agreement scheme for e-healthcare cloud systems, we first state the underlying computational problem and assumption as follows:

### Decisional Diffie-Hellman (DDH) problem [39]

Let *p* and *q* be two large primes satisfying *q* | *p*– 1, and *g* a generator of order *q* over GF(*p*). The DDH problem is, given (*p*, *q*, *g*^*a*^, *g*^*b*^) and *g*^*c*^ for some *a*, *b*, *c* randomly and independently chosen from *Z*_*q*_, to decide whether *g*^*c*^ = *g*^*ab*^ mod *p* or not.

### Decisional Diffie-Hellman (DDH) assumption [39]

The advantage for any probabilistic polynomial-time (PPT) algorithm *A* to solve the DDH problem is negligible.

When it comes to user authentication protocols, we usually consider the crucial security requirement of authenticated key exchange (AKE). In this security notion, an adversary A can make **Test** queries to obtain either an invalid symbol ⊥ or certain valid value. The former case happens when a protocol **P** rejects a user instance. In the latter case, the valid value could be a real session key if such a **Test** query is made in relation to partnered instances and only one of them is honest. If not, the query will return either the genuine session key or a random number depending on the result of a flipped internal bit *b*. At last, the adversary A outputs a guessed bit *b'*. We say that A wins the indistinguishability game against the protocol **P** only if *b* = *b'*. The advantage of adversary A against the protocol **P** could be defined as $Adv_{P}{}^{ake}(A)=\left|\mathrm{Pr}[b=b^{\prime }]-\frac{1}{2}\right|$. When $Adv_{P}{}^{ake}(A)$ is negligible, we can claim that the protocol **P** is AKE-secure.

In practical application environments, a patient *ID*_*i*_ and an e-healthcare cloud server $ID_{S_{j}}$ might be participated in various concurrent connections by using unique session keys, respectively. We therefore use the notation of ($\Pi_{U_{i}}^{m}$, $\Pi_{S_{j}}^{n}$) to represent an instance (also known as oracle) of players (*ID*_*i*_, $ID_{S_{j}}$) engaged in the (*m*, *n*)-th session, respectively. In general, a malicious adversary A is allowed to invoke the following queries:

- ***Send***($\Pi_{U_{i}}^{m}$, *M***)**: A ***Send*** query enables the adversary to take control of interactive communications of a protocol **P**. More precisely, a ***Send***($\Pi_{U_{i}}^{m}$, *M*) query gives A the computational results of the instance $\Pi_{U_{i}}^{m}$ with respect to the message *M* according to **P**’s protocol steps. Additionally, the ***Send***($\Pi_{U_{i}}^{m}$, “*start*”) query indicates $\Pi_{U_{i}}^{m}$ to initialize the execution of protocol **P**.
- ***Corrupt*(***U***)**: By issuing this query, the adversary *A* can acquire the long-term private key of user *U*. In the proposed system, the session keys are viewed as short-term private keys while the passwords of users are thus regarded as long-term ones. This query can model the security requirement of perfect forward secrecy, i.e., a protocol **P** can still guarantee the confidentiality of previous short-term private keys even if the long-term ones have been exposed.
- ***Hash*(***M***)**: This query models the hash functions *H*_1_(•) and *H*_2_(•) of the proposed system. The adversary can make a ***Hash***(*M*) query and receive the generated result. If no matched entry can be found out in the target list, the oracle randomly selects a value *a* of proper length to return. The entry (*M*, *a*) is also preserved in the list.
- ***Reveal***($\Pi_{U_{i}}^{m}$): Once an instance $\Pi_{U_{i}}^{m}$ is accepted by a protocol **P**, *A* can invoke a ***Reveal***($\Pi_{U_{i}}^{m}$) query to learn its real session key. Otherwise, the oracle returns **Fail**. Such a query is used to evaluate the confidentiality of session keys when one of them is compromised, and is thus referred to as known key attacks.
- ***Test***($\Pi_{U_{i}}^{m}$): To simulate the security requirement for indistinguishability of session key *SK*, *A* can make use of ***Test*** queries. When a ***Test***($\Pi_{U_{i}}^{m}$) query is invoked and neither $\Pi_{U_{i}}^{m}$ nor its partner has been queried the ***Reveal*** oracle, it will return the real session key *SK*_*i*_. Still, in another fresh case that both $\Pi_{U_{i}}^{m}$ and its partner are finally accepted by the protocol **P**, and on one had been issued the ***Reveal*** oracle, it will flip an internal coin *b* to decide a returned value. If *b* = 1, the real session key *SK*_*i*_*'* is outputted; else if *b* = 0, a random number *SK*_*i*_*'* of the same length is returned instead.

To prove the AKE-security of the proposed protocol, we first recall the definition of Difference Lemma [40] as follows:

### Lemma 1. Difference lemma

Let *A*, *B* and *F* be events in some probability distribution. If the condition that Pr[*A* ∧ ¬*F*] = Pr[*B* ∧ ¬*F*] holds, we can derive | Pr[*A*]–Pr[*B*] | ≤ Pr[*F*].

From the perspective of theoretic security, we will formally prove that our protocol satisfies the AKE security in the random oracle model by employing the method of sequence of games along with the Difference Lemma as Theorem 1.

**Theorem 1.** *Let Adv*^*ddh*^ denote *the advantage of a DDH adversary who has the ability to break the DDH problem within the running time t*. *Then we could express the advantage for an adversary breaking the AKE security of proposed protocol* ***P*** *as*

$$Adv_{P}{}^{ake}(t^{\prime },q_{s},q_{H})\leq \frac{q_{s}}{\omega }+\frac{q_{H}{}^{2}}{2^{k}}+Adv^{ddh}(t,q_{s},q_{H}),$$

*where q*_*s*_ *and q*_*H*_ *separately represent the number of* ***Send*** *and* ***Hash*** *queries*, *and the symbol* ω *is the dictionary size of passwords*.

**Proof:** The proof idea is as follows. We first construct a sequence of games, named *G*_*i*_’s, for *i* = 0 to 4. In each game *G*_*i*_, an adversary wins the game is defined as an event *E*_*i*_. The transition between consecutive games, i.e., from *G*_*i*_ to *G*_*i*+1_ is made by adding slight modifications. We shall show that the difference between Pr[*E*_*i*_] and Pr[*E*_*i*+1_] is negligible. Let game *G*_0_ be an adversary A attempting to defeat the AKE security of proposed protocol **P** in the real world. Since we have derived that Pr[*E*_*i*_] = Pr[*E*_*i*+1_] for *i* = 0 to 3, it is sufficient to claim that Pr[*E*_0_] approximates Pr[*E*_4_] which is negligible. Consequently, we could complete this security proof with the final game *G*_4_ showing that it is negligible for any probabilistic polynomial-time algorithm to defeat the AKE security of protocol **P**.

**Game *G***_**0**_: This game models a real situation that an adversary A tries to break the semantic security of session key *SK* in the proposed protocol **P**. More specifically, A will invoke a ***Test*** query and output a bit *b'*. When *b* = *b'*, which is defined as the event *E*_0_, A wins the indistinguishability game against the protocol **P**. According to previous definition, we learn that

$$Adv_{P}{}^{ake}(A)=\left|\mathrm{Pr}[E_{0}]-\frac{1}{2}\right|.$$ (26)

**Game *G***_**1**_: This game simulates the scenario that an adversary A aims at guessing a correct password, i.e., long-term private key of some user instance $\Pi_{U_{i}}^{m}$ by invoking ***Send*** queries. Nevertheless, a ***Send*** query of message *M'* composed of (*u*, *d*_*i*_, *z*, *t*_1_) will lead to different computational results during each execution of protocol **P**. Namely, it would be intractable for A to verify his/her guess. Hence, the success probability of this game could be expressed as the event that a ***Send*** query consisting of valid message *M'* = (*u*, *d*_*i*_, *z*, *t*_1_) has been invoked, denoted by *E*_1_. Then, we can compute

$$\left|\mathrm{Pr}[E_{0}]-\mathrm{Pr}[E_{1}]\right|\leq \frac{q_{s}}{\omega }.$$ (27)

**Game *G***_**2**_: In this game, we keep a list for correctly responding to all ***Hash*** queries. As long as no collision for each ***Hash*** query is found out by the adversary, the game is perfectly simulated just like previous game *G*_1_. We therefore define *E*_2_ to be the event of some hash collision happening in the simulation. Then, by employing the Difference Lemma and the birthday paradox, we know that

$$\left|\mathrm{Pr}[E_{1}]-\mathrm{Pr}[E_{2}]\right|\leq \frac{q_{H}{}^{2}}{2^{k}}.$$ (28)

**Game *G***_**3**_: We made a transition of game *G*_2_ to game *G*_3_ by adding a simulator S. The simulator S acts based on game *G*_2_ to simulate all oracles except that the ***Send*** queries composed of a random DDH triple (u*, *Y*_j_*, w*) for honest players would be replaced by another indistinguishable triple (*X*, *Y*, *Z*). First, the simulator S randomly chooses a*, *x*_j_* ϵ *Z*_*q*_, sets *S*_*j*_’s private key as *x*_j_*, computes *X* = *g*^a*^ mod *p*, *Y* = *g*^xj*^ mod *p* and *Z* = (*Y*)^a*^ mod *p*, and then records the entries (a*, *X*), (*x*_j_*, *Y*) and (*X*, *Y*, *Z*). By doing so, the simulator S is able to correctly respond to all the ***Send*** and ***Test*** queries in game *G*_3_. It is evident that we utilize computationally indistinguishable DDH triples to perfectly substitute for random DDH triples in game *G*_2_. Consequently, the success probability of an adversary in game *G*_3_ is determined by the event probability of *E*_3_, i.e., distinguishing a random DDH triple and a simulated one computed by the simulator S, and we can observe that

$$\mathrm{Pr}[E_{2}]=\mathrm{Pr}[E_{3}].$$ (29)

**Game *G***_**4**_: We start the final game and simulate all the oracles just like what we have done in game *G*_3_. Yet, we add some little changes by using identically distributed random variables (X*, Y*, Z*) to substitute for the computationally indistinguishable DDH triple (*X*, *Y*, *Z*) of related oracles. Assume that there is a polynomial-time adversary D trying to solve the above instance of DDH problems within the running time *t*. The adversary D flips an internal coin *b* to decide how it interacts with A. Whenever *b* = 1, the real session key *SK* is outputted to A. Otherwise, a random variable of the same length is returned to A instead. At last, A will generate a bit *b'* as its guess. Only when the equality *b'* = *b* holds, we say that A wins the indistinguishability game. At the same time, D would finally output 1; else, D outputs 0. We express the event that D finally returns 1 as *E*_4_ and we have

$$\mathrm{Pr}[E_{4}]=\frac{1}{2}$$ (30)

if the triple (X*, Y*, Z*) is truly random variables and no information about the bit *b* is leaked. On the other hand, if the random triple (X*, Y*, Z*) is also a DDH triple, Pr[*E*_4_] would be equivalent to Pr[*E*_3_] and we could derive that

$$\left|\mathrm{Pr}[E_{3}]-\mathrm{Pr}[E_{4}]\right|\leq Adv^{ddh}(D),$$ (31)

Combining Eqs (26) to (31), we have that

$$Adv_{P}{}^{ake}(A)=\left|\mathrm{Pr}[E_{0}]-\frac{1}{2}\right|$$

$$\leq \frac{q_{s}}{\omega }+\frac{q_{H}{}^{2}}{2^{k}}Adv^{ddh}(D),\;\mathrm{which}\;\mathrm{is}\;\mathrm{negligible}.$$ Q.E.D.

**Fig 4:**
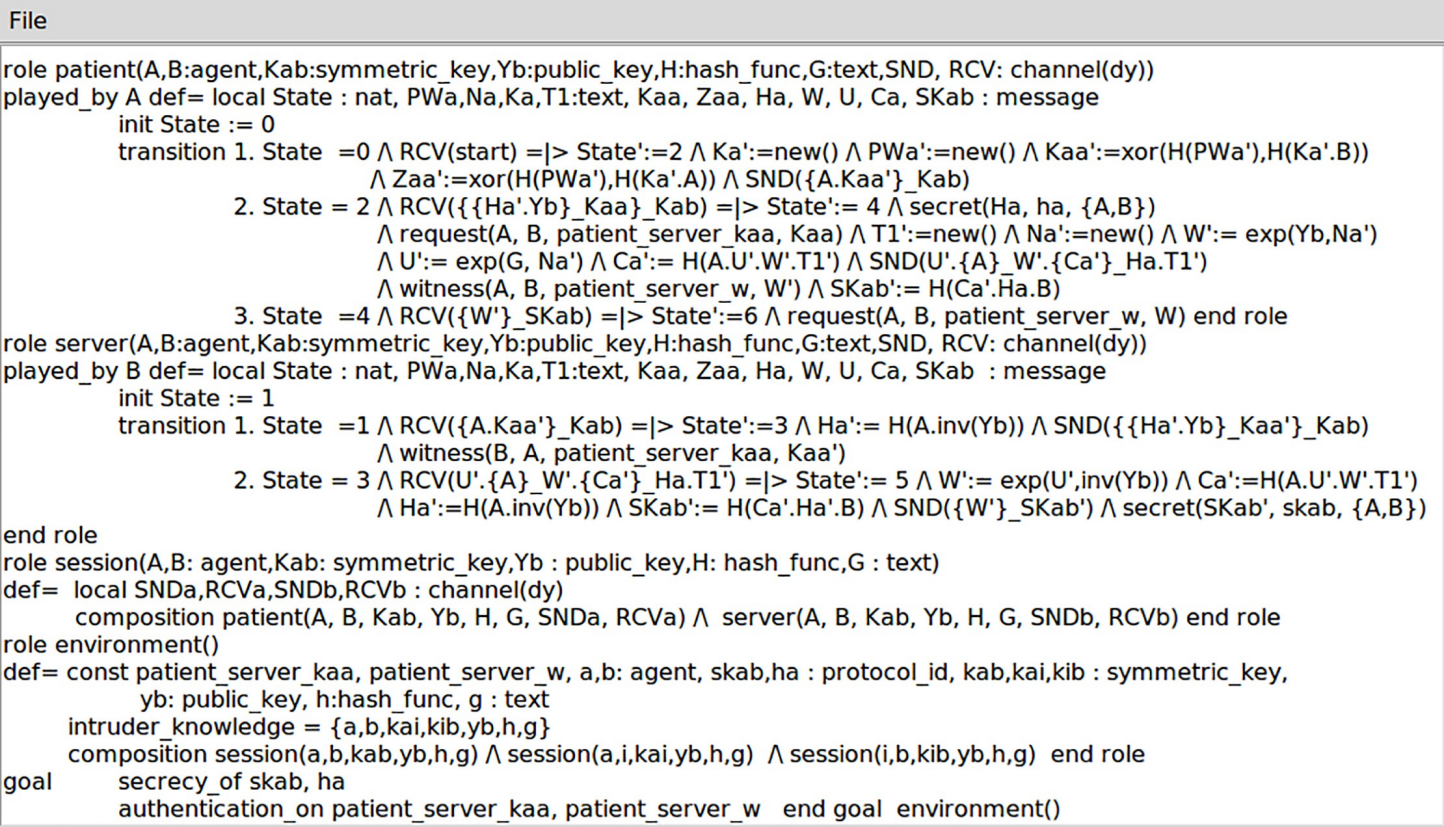
HLPSL specifications of our scheme.

**Fig 5:**
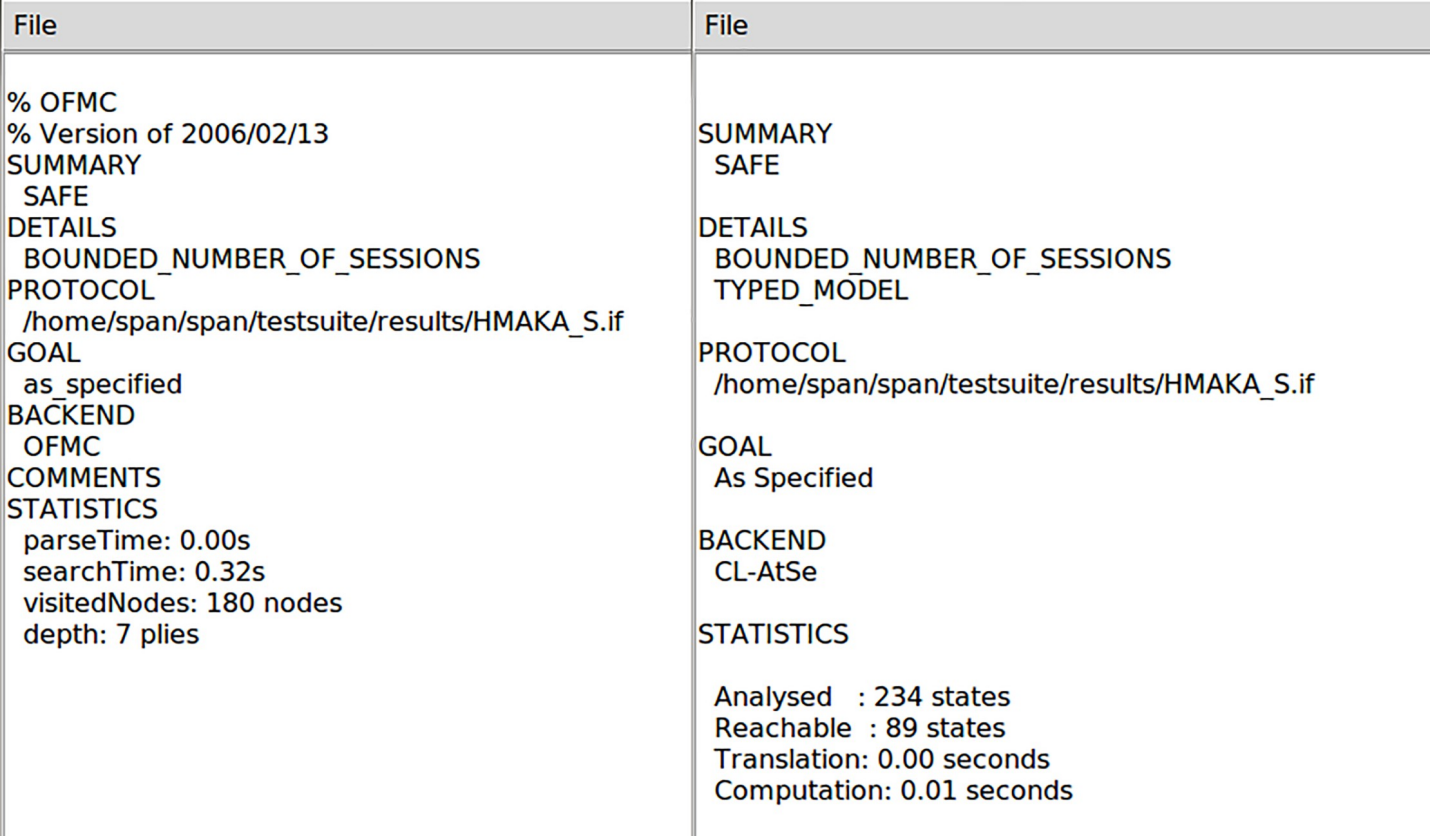
Analysis results of OFMC and CL-AtSe modules.

We further evaluate the security of our scheme by utilizing the well-developed tool of AVISPA (Automated Validation of Internet Security Protocols and Applications) [41]. Such a security analysis tool integrates several back-ends (analyzers) to realize automatic validation of security protocols as well as tracing possible attacks against the designed cryptographic schemes. Concretely speaking, the AVISPA has four modules including OFMC (On-the-Fly Model-Checker), CL-AtSe (Constraint-Logic-based Attack Searcher), SATMC (SAT-based Model-Checker) and TA4SP (Tree Automata tool based on Automatic Approximations for the Analysis of Security Protocols). Any security protocol to be analyzed must be specified in the format of HLPSL (High Level Protocols Specification Language) which will be transformed to IF specifications by a translator called hlpsl2if. Fig 4 illustrates the HLPSL specifications of our scheme. Then we employ the OFMC and the CL-AtSe modules to evaluate the security of our protocol. The analysis results shown in Fig 5 both reveal “SAFE” for our proposed protocol.

## Comparison

In this section, we compare our authentication scheme with the Yang-Yang (YY for short) [32], the Khan-Kumari (KK for short) [42] and Chen *et al*.’s (CKW for short) [17] mechanisms in terms of security features and computational efforts. For facilitating the comparison, we first define some used symbols as Table 2. The approximate running time of each evaluated operation is also simulated according to [43, 44]. The detailed comparisons are listed in Table 3. From this table, one can observe that all compared protocols fail to provide user anonymity and provable AKE security. The YY scheme has to further assume a trusted third party (TTP). The KK scheme is the most time-consuming in the authentication processes. As for the CKW scheme, it not only incurs high computation overheads, but also cannot fulfill the requirements of offline password update, key contributory property and perfect forward secrecy. Here, we would like to discuss some aspects of the Man-At-The-End (MATE) attack [45] which is originated from the applications of digital assets’ protection (DAP) and software protection. Fundamentally, such an attack is very difficult to evaluate and analyze than other security requirements due to its various forms and the complicated human nature. In a commonly seen scenario of MATE attacks, an adversary obtaining the access privilege to physical hardware might attempt to tamper it or inspect contained software. Consequently, we must be aware of the impact of MATE attacks and strengthen our current fortifications by resorting to anti-tamper techniques and software protection mechanisms. To sum up, the proposed scheme is still a better alternative from the perspective of functionalities, security and computational efficiency.

**Table 2:** The used notations.

	Computation	Approximate running time
**H**	Collision-resistant hash function	0.2ms
**M**	Modular multiplication	0.2ms
**E**	Modular exponentiation	48ms

**Table 3:** Computational comparisons of the proposed and previous schemes.

	YY	KK	CKW	Ours
**Anonymity**	No	No	No	Yes
**Mutual authentication**	Yes	Yes	Yes	Yes
**Resist man-in-the-middle attack**	Yes	Yes	Yes	Yes
**Resist man-at-the-end attack**	Unknown	Unknown	Unknown	Unknown
**Offline password update**	Yes	Yes	No	Yes
**Key contributory property**	Yes	Yes	No	Yes
**Without trusted third party (TTP)**	No	Yes	Yes	Yes
**Perfect forward secrecy**	Yes	Yes	No	Yes
**Provable AKE security**	No	No	No	Yes
**Computational cost for** **registration**	E + 3H (≈ 48.6ms)	E + 4H (≈ 48.8ms)	E + H (≈ 48.2ms)	4H (≈ 0.8ms)
**Computational cost for authentication**	4E + 8H (≈ 193.6ms)	5E + 9H (≈ 241.8ms)	3E + 3M + 8H (≈ 146.2ms)	3E + 7H (≈ 145.4ms)
**Computational cost for password-update**	3H (≈ 0.6ms)	6H (≈ 1.2ms)	2E + 2M + 2H (≈ 96.8ms)	3H (≈ 0.6ms)
**Total computational cost**	5E + 14H (≈ 242.8ms)	6E + 19H (≈ 291.8ms)	6E + 5M + 11H (≈ 291.2ms)	3E + 14H (≈ 146.8ms)

## Conclusions

To provide a secure mechanism for accessing the resources of e-healthcare cloud systems implemented in a PKI, we propose a new heterogeneous mobile authentication and key agreement scheme using security token hardware. Our scheme preserves the property of user anonymity and allows users to change their passwords without the intervention of remote cloud servers. Each user can solely update his password with the help of his security token device. Besides, a remote e-healthcare cloud server is unnecessary to keep a password table for authenticating users, so as to prevent the risk of information leakage. Based on the security assumption of DDH which is believed to be polynomial-time intractable, we formally proved that our scheme achieves the AKE-security in the random oracle model. As for the practical application security, the well-developed AVISPA security protocol validation tool also found no possible attacks or pitfalls in the designed mechanism. Moreover, we demonstrate that the proposed scheme owns better security features and takes lower computational costs.
